# Measurement Campaign of Radio Frequency Interference in a Portion of the C-Band (4–5.8 GHz) for the Sardinia Radio Telescope

**DOI:** 10.3390/s24196481

**Published:** 2024-10-08

**Authors:** Luca Schirru, Francesco Gaudiomonte

**Affiliations:** National Institute for Astrophysics (INAF), Cagliari Astronomical Observatory, Via della Scienza 5, 09047 Selargius, Italy; francesco.gaudiomonte@inaf.it

**Keywords:** radio astronomy, radio frequency interference (RFI), C-band, Sardinia Radio Telescope (SRT)

## Abstract

Radio frequency interference (RFI) analysis is crucial for ensuring the proper functioning of a radio telescope and the quality of astronomical observations, as human-generated interference can compromise scientific data collection. The aim of this study is to present the results of an RFI measurement campaign in the frequency range of 4–5.8 GHz, a portion of the well-known C-band, for the Sardinia Radio Telescope (SRT), conducted in October–November 2023. In fact, this Italian telescope, managed by the Astronomical Observatory of Cagliari (OAC), a branch of the Italian National Institute for Astrophysics (INAF), was recently equipped with a new C-band receiver that operates from 4.2 GHz to 5.6 GHz. The measurements were carried out at three strategically chosen locations around the telescope using the INAF mobile laboratory, providing comprehensive coverage of all possible antenna pointing directions. The results revealed several sources of RFI, including emissions from radar, terrestrial and satellite communications, and wireless transmissions. Characterizing these sources and assessing their frequency band occupation are essential for understanding the impact of RFI on scientific observations. This work provides a significant contribution to astronomers who will use the SRT for scientific observations, offering a suggestion for the development of mitigation strategies and safeguarding the radio astronomical environment for future observational campaigns.

## 1. Introduction

Radio astronomy has been instrumental in advancing our understanding of the universe, providing crucial insights into cosmic phenomena ranging from the structure of galaxies to the discovery of exoplanets. However, the increasing prevalence of man-made signals, named radio frequency interference (RFI), poses a significant threat to the sensitivity and accuracy of radio telescopes during scientific observations. RFI originates from sources installed on the territory surrounding the telescope, such as terrestrial wireless networks and everyday electronic devices, or from space-to-ground transmissions including communication satellites [1,2]. In addition, a considerable number of undesired signals can be self-produced by the telescope through its electronic devices, such as digital back-ends, control systems, etc. [3]. Mitigating RFI is vital for the success of radio astronomy and its scientific accuracy. The signals that radio telescopes aim to detect are often extremely weak, requiring highly sensitive equipment that is also vulnerable to external noise. This vulnerability is exacerbated by the rapid growth of wireless communication technologies, which has resulted in a crowded radio spectrum. Consequently, radio astronomers are increasingly confronted with the task of distinguishing between genuine astronomical signals and spurious RFI, a process that is both time-consuming and technically challenging.

In response to these challenges, various techniques have been developed to detect, mitigate, and manage RFI in radio telescopes. These techniques include time and frequency filtering (i.e., microwave filters), adaptive beamforming, and advanced signal processing algorithms [3,4,5,6,7,8,9]. Additionally, it is necessary to periodically conduct specific measurement campaigns to monitor the RFI environment surrounding the telescope. Such measurements are essential for implementing appropriate mitigation procedures [10,11,12,13].

Furthermore, regulatory frameworks at both national and international levels have been established to allocate and protect certain frequency bands for radio astronomy. The International Telecommunication Union Radio Regulations (ITU-RR) assigned specific windows of the entire frequency spectrum to the radio astronomy service (RAS) with the aim of ensuring that sensitive observations are not compromised by interference from other radio frequency emissions [14]. Radio receivers for radio telescopes are designed with wide bandwidths that also include the RAS bands. In this way, the analysis of the spectrum can occur in a larger field than the narrow range of the RAS bands at the cost of detecting a considerable amount of RFI.

Focusing attention on the so-called C-band, generally defined as the frequency range from 4 GHz to 8 GHz, this is a vital portion of the electromagnetic spectrum used by various services, including satellite communications, radar, and wireless broadband. Within this range, the primary RAS frequencies are 4.8–5.0 GHz and 6.65–6.6752 GHz [14]. These bands are crucial for studying celestial sources such as molecular clouds, galaxies, and cosmic microwave background radiation. Because the signals generated by these sources are so weak, the RAS frequencies in the C-band are particularly vulnerable to interference from terrestrial communication systems and other man-made sources. As a result, the protection and careful management of these frequencies are essential to maintain the integrity of astronomical research, enabling scientists to continue making groundbreaking discoveries in our understanding of the universe.

In Italy, the Sardinia Radio Telescope (SRT), managed by the Astronomical Observatory of Cagliari (OAC), a branch of the Italian National Institute for Astrophysics (INAF), is a sophisticated instrument capable of operating across a broad frequency range, from 300 MHz to 116 GHz [15]. Among the various cryogenic receivers installed on the telescope, one operates in the 4.2–5.6 GHz window of the C-band and is called the C-low receiver [16]. This receiver was installed in October 2023 and has been invaluable from the outset for very long baseline interferometry (VLBI) observations, as well as for various scientific projects, with the data still being processed. The SRT is situated in the Pranu Sanguini area, approximately 35 km northeast of Cagliari (latitude 39.493072° N, longitude 9.245151° E) at an elevation of about 650 m above sea level. Detailed descriptions of the telescope’s technical specifications, including its radio receiver features, are provided in [17,18].

This paper presents the methodology and results of an RFI measurement campaign conducted in the range of 4–5.8 GHz. The measurements were carried out in the area surrounding the site of the SRT during October and November of 2023. Thanks to the results of this measurement campaign, it is possible to map the RFI scenario in the C-band portion used by the SRT’s cryogenic receiver. This facilitates the processing of data collected during scientific observations by filtering out spurious signals from the surrounding environment. Additionally, based on the campaign’s findings, preventive measures can be implemented to enhance the observation experience. For example, adjusting the local oscillator to a specific frequency can help filter out unwanted portions of the band. Section 2 provides a detailed description of the measurement setup. The results of the RFI measurement campaign are presented in Section 3. Lastly, Section 4 offers a discussion and conclusions based on the findings.

## 2. Materials and Methods: Description of the Measurement Setup

The RFI measurement campaign was conducted using the INAF mobile laboratory around the SRT site in October–November 2023. Specifically, three strategic geographic points were selected to ensure comprehensive coverage of the entire area surrounding the telescope. The measurement scenario is described in Section 2.1, while the mobile laboratory is detailed in Section 2.2.

### 2.1. Description of the Measurement Scenario

**Figure 1:**
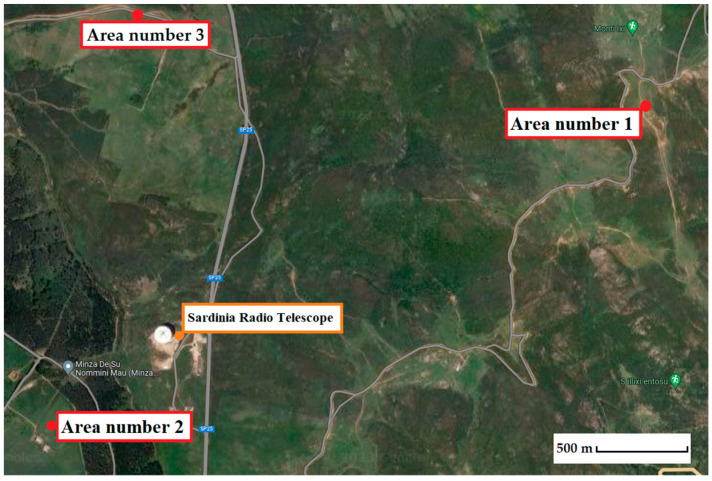
Map of the selected points for the RFI measurement campaign.

The three areas selected for the measurement campaign are located at altitudes comparable to the position where the C-band receiver of the SRT is installed. This approach ensures that each antenna pointing direction at low elevation angles (i.e., down to 5 degrees) is covered, and the results presented in this paper correspond to all signals detectable during operational sessions. The three selected locations are shown in the map in Figure 1 and have the following geographic characteristics:

- Area number 1: a point on Monte Ixi mountain (lat. 39.50075958202559° N, long. 9.26616912945711° E), located approximately 2 km from the SRT as the crow flies;
- Area number 2: a point in the Union of Municipalities of Gerrei, called Colonia Montana (lat. 39.4900647290649° N, long. 9.23939203600374° E), located at about 600 m from the SRT as the crow flies;
- Area number 3: a point on the pasture land in front of the telescope (lat. 39.503814094404156° N, long. 9.245117265226954° E), placed approximately 1 km from the SRT as the crow flies.

The measurements were conducted in stable weather conditions, with clear skies and variable winds, where average gusts did not exceed 15 km/h.

### 2.2. Description of the INAF Mobile Laboratory

The INAF mobile laboratory allows measurements over a wide frequency band, ranging from 250 MHz to 40 GHz [19]. It is equipped with a retractable telescopic mast on which the antenna is mounted. The antenna can be rotated manually or electronically in the azimuthal direction via a motor. A second motor at the top of the mast allows for the rotation of the antenna to select either horizontal or vertical polarization.

**Figure 2:**
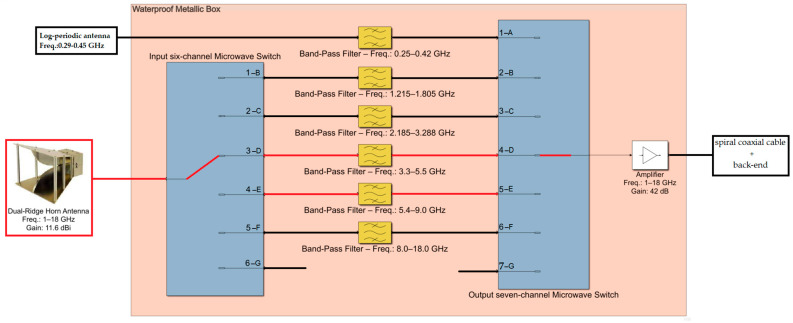
The front end of the INAF mobile laboratory is composed of six radio frequency channels, covering the entire frequency range from 250 MHz to 40 GHz. The channels highlighted in red (i.e., channels D and E) were used for the measurement campaign presented in this paper.

Concerning the front end, the INAF mobile laboratory is equipped with six different radio frequency paths (i.e., channels) for operations up to 18 GHz, as well as an additional configuration for measurements from 18 GHz to 40 GHz [19]. A block diagram of the front end, operating up to 18 GHz, is shown in Figure 2. Each path is composed of commercial microwave components optimized for a specific frequency range, for which an appropriate antenna can be chosen from the five available. A detailed description of the entire front end, including the total characterization of each microwave component and antenna model, is provided in [20]. In particular, channels D and E, highlighted in red in Figure 2, are used for the C-band measurements presented in this paper.

For the measurements presented in this work, a dual-ridge horn antenna, model DRG-118-A from A.R.A. Technologies (Laurel, MD, USA) [21], was used. The antenna has an average gain of 11.6 dBi and an average antenna factor of 36.6 dB/m within the frequency range of 1–18 GHz. Analyzing Figure 2, the antenna is connected via a coaxial cable to a waterproof metallic box housing the microwave components of the front end. The first component of this box is a six-channel microwave switch, model RLC SR-6C-H from RLC Electronics (Mount Kisco, NY, USA) [22], which has an insertion loss of approximately 0.3 dB and channel isolation of approximately 60 dB in the C-band. A microwave filter is directly connected to the switch. Specifically, for channel D, a band-pass cavity filter, model 8IZ5-4400/R2200-S from Lorch (Cedar Hill, TX, USA) [23], with an insertion loss of less than 1 dB in the 3.3 GHz to 5.5 GHz range, is used. For channel E, a band-pass cavity filter, model 8IZ6-7200/R3600-S from Lorch [23], with an insertion loss of less than 1 dB in the 5.4 GHz to 9 GHz range, is employed. For each of the two channels, the filter is connected in series with a low-pass filter, which ensures the suppression of frequencies above the cutoff. Another switch, a seven-channel microwave switch, model RLC SR-7C-H from RLC Electronics [22], with similar characteristics to the input switch described above, is installed for ending the multiple channel selection. This output switch is connected via a coaxial cable to an amplifier, model AMF-6D-00101800-35-20P from Miteq (Hauppauge, NY, USA) [24], which has an average gain of approximately 42 dB, an output 1 dB compression point of +20 dBm, and a noise figure of 3.5 dB over the frequency range from 100 MHz to 18 GHz. In addition to these radio frequency components, an electronic board for powering the active devices is installed inside the metallic box.

The front end is connected via a coaxial cable to an external spiral coaxial cable, approximately 17 m in length, which wraps around the retractable telescopic mast of the mobile laboratory and facilitates the connection with the laboratory’s back end. This spiral cable has a loss of approximately −15 dB at 5 GHz.

A summary of the technical specifications (i.e., frequency range and average gain at 5 GHz) for each component of the receiving chain is reported in Table 1.

The back end of the laboratory includes a spectrum analyzer, model FSV40 from Rohde & Schwarz, Columbia, IN, USA [25], and a professional telecommunication receiver, model IC-R9500 from ICOM, Kirkland, WA, USA [26]. These instruments are installed inside the van and enable the comprehensive characterization of detected signals, identifying key features such as amplitude, central frequency, bandwidth, polarization, and modulation type.

## 3. Results and Discussion

The measurement campaign, conducted in October–November 2023, focuses on both impulsive and stationary signals, aiming to detect the various types of signals propagating in the air. To differentiate between them, the spectrum analyzer was configured by appropriately setting the resolution bandwidth (RBW) of the instrument. Specifically, for continuous signals, the RBW was set to a few hundred kHz (e.g., 300 kHz), which is why this is referred to as the “narrow-band campaign”. In contrast, detecting impulsive signals requires a wider RBW, typically a few MHz (e.g., 3 MHz), which is why the measurement of impulsive signals is called the “wideband campaign”.

The mechanical measurement procedure involves several slow 360-degree rotations of the mobile laboratory’s mast, where the antenna is mounted. The entire process lasts approximately 15 min, which is considered sufficient to map the general RFI scenario in the area of interest. During these antenna rotations, the spectrum analyzer is set to “max hold” mode, and the data are recorded. This process is repeated for each linear polarization of the antenna (i.e., horizontal and vertical), and the data are then combined. This approach ensures that all azimuthal directions are considered, allowing for an accurate mapping of the surrounding RFI environment.

The amplitude level of the measured signals, since it does not cause compression of the components in the mobile laboratory’s receiving chain, is not relevant to this study. In fact, the C-low receiver of the SRT is a more robust system than the mobile laboratory’s front end, and there is no risk of it being saturated. The purpose of this study is to assess the frequency band occupancy of the unwanted signals detectable by the telescope.

**Figure 3:**
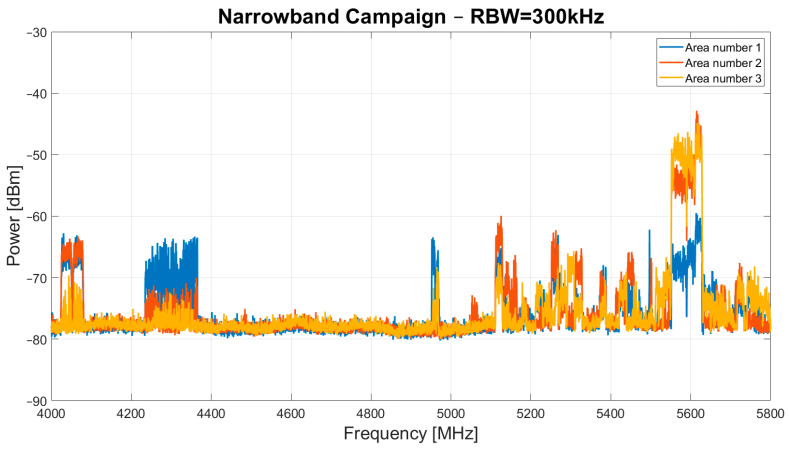
Results of narrowband campaign, with the RBW of the spectrum analyzer set to 300 kHz, conducted in October–November 2023 in area number 1, area number 2 and area number 3.

**Figure 4:**
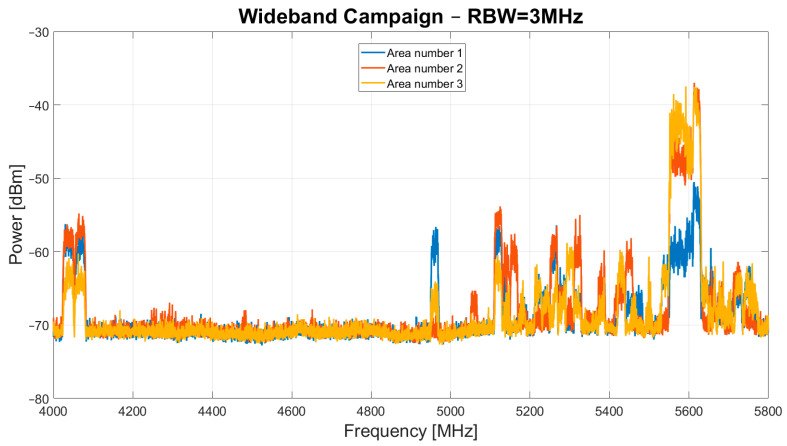
Results of wideband campaign, with the RBW of the spectrum analyzer set to 3 MHz, conducted in October–November 2023 in area number 1, area number 2 and area number 3.

The results of the narrowband and wideband campaigns are presented in Figure 3 and Figure 4, respectively.

Regarding the narrowband campaign, Figure 3 shows a high presence of in-band signals in the 4.03–4.08 GHz, 4.23–4.37 GHz, 4.95–4.97 GHz, and 5.1–5.8 GHz frequency ranges. These signals are generally detected in all three measurement areas. However, in area number 2, signals were detected at 5.05 GHz that were not present in the other two areas.

The most notable finding is that the 4.37–4.95 GHz window has the least RFI, and this frequency window is the most suitable for scientific applications.

As for the results of the wideband campaign, shown in Figure 4, the same band occupancy is observed, except for the signals in the 4.23–4.37 GHz frequency range. These signals are evidently not detectable with the spectrum analyzer set to an RBW of 3 MHz and therefore are not attributable to the signals targeted by this campaign.

**Figure 5:**
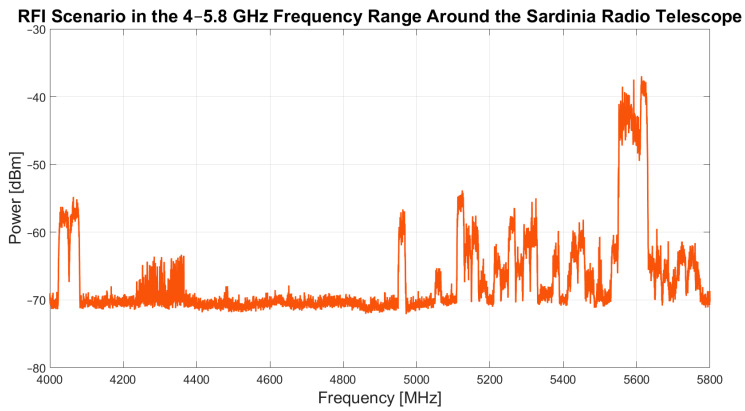
Summary chart of the entire RFI scenario in the frequency range from 4 to 5.8 GHz around the Sardinia Radio Telescope (SRT). This chart includes all signals detected during both the narrowband and wideband campaigns, conducted in October–November 2023.

Finally, a summary chart of the entire band occupancy, including both detected continuous and impulsive signals, is shown in Figure 5. This chart is crucial for astronomers using the SRT, as it provides a reference for the band occupancy of the C-low receiver by signals from the telescope’s surrounding environment, which can be detected with every pointing direction of the telescope.

**Figure 6:**
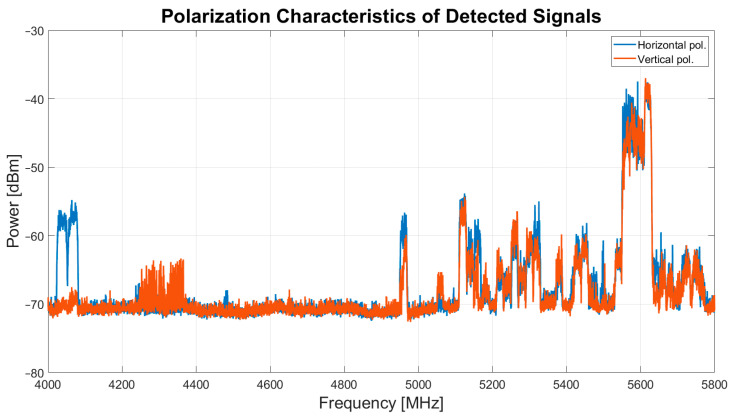
Polarization characteristics of detected signals during both the wideband and narrowband campaigns, conducted in October–November 2023.

For a more detailed analysis of the RFI scenario, Figure 6 presents a graph that distinguishes between signals detected with the antenna polarized horizontally and vertically. It is noteworthy that except for the signals in the frequency range of 4.03–4.08 GHz, which were detected only during measurements with horizontal polarization, all other signals were detected with both antenna polarizations. This differentiation of RFI polarization can be useful for astronomers when planning scientific observations with the SRT.

For informational purposes, an analysis of the state of the art reveals that the signals detected during this measurement campaign can be traced back to satellite communications, including full-time satellite TV networks or raw satellite feeds [27,28,29], as well as to terrestrial wireless broadband systems used for both civil and military communications [30] and several radar applications [31].

Based on the results in Figure 5, scientific observations can be optimized by implementing various RFI mitigation techniques, such as using software to remove unwanted signals or shifting to an interference-free frequency range by appropriately adjusting the local oscillator. Indeed, the SRT operates as a super heterodyne system, with most of its receivers featuring a frequency down-conversion system that shifts the detected signals from the sky to a baseband frequency range of 100 MHz to 2.1 GHz. For the C-low receiver (a detailed description of the system is provided in [16]), which covers a bandwidth from 4.2 GHz to 5.6 GHz, a filter at an intermediate frequency (for example, from 100 MHz to 400 MHz) within the telescope’s receiving chain can be used to isolate a specific portion of the receiver’s bandwidth. By adjusting the local oscillator frequency of the receiver, directly settable from the SRT control system, it is possible to select the desired free-of-RFI bandwidth segment from 4.37 to 4.95 GHz that will coincide to the actual bandwidth of the filter at the intermediate frequency.

The results presented in this article could be refined by scheduling a new measurement campaign using both the telescope directly and the mobile laboratory. This would involve conducting scans in all azimuthal directions while varying the elevation angle to determine the exact direction of the source relative to the telescope’s position. Such an approach could allow for the creation of a detailed three-dimensional map of the sources generating RFI. Consequently, it would help identify the telescope’s pointing directions that would lead to receiving the strongest signals. This type of work could be valuable for filtering out unwanted signals during survey-mode observations, which involve continuous scanning of the sky for radio astronomical sources.

## 4. Conclusions and Future Work

This paper presents the measurement setup and results of an ad hoc campaign aimed at detecting unwanted signals in a portion of the C-band (4–5.8 GHz) around the SRT site. The goal is to provide astronomers with a comprehensive overview of the RFI scenario detectable during SRT observations using its C-low receiver, which operates in the frequency range of 4.2–5.6 GHz. To achieve this, three strategically chosen geographic points were selected for the measurement setup to cover the entire area surrounding the telescope at altitudes comparable to the position where the C-low receiver is installed on the SRT. This approach ensures that every antenna pointing direction at low elevation angles is covered. The results highlight a significant presence of RFI at both the beginning and end of the examined C-band portion (4–5.8 GHz). Thanks to the results of this work, astronomers can implement a range of mitigation procedures during observations and data reduction, such as removing RFI with appropriate software or configuring the telescope to observe in RFI-free areas of the band.

As a future work, a new measurement campaign could be planned to detect all unwanted signals self-generated by the control electronics of the equipment within the telescope. This equipment may include radio astronomy receivers, control systems for the movement of the telescope’s mirrors, and more. The campaign will be conducted directly inside various sections of the antenna, aiming to identify the devices responsible for generating these unwanted signals and to implement mitigation procedures. Additionally, the measurement campaign presented in this paper could be repeated periodically, for example, once a year or every two years, to provide users of the telescope with an up-to-date RFI scenario. Furthermore, a new measurement campaign could be conducted using both the SRT and the mobile laboratory to facilitate a more detailed study of the detected RFI signals, including their amplitudes. This would involve employing radio astronomy back ends and presenting the data in formats typical of scientific observations. Finally, a real-time monitoring system for RFI could be implemented to provide added value to astronomers during scientific observations.

In conclusion, the work presented in this article, along with the proposed future developments, can be extended to all frequency ranges covered by the SRT receivers.

## Figures and Tables

**Table 1:** Summary of the technical specifications for each component of the receiving chain.

Channel of the Front End	Component of the Receiving Chain	Frequency [GHz]	Gain at 5 GHz [dB]
**Common for all channels**	Dual-ridge horn antenna, model DRG-118-A from A.R.A. Technologies	1–18	11.6
D	Input switch, model RLC SR-6C-H from RLC Electronics	0–18	−0.3
	Band-pass cavity filter, model 8IZ5-4400/R2200-S from Lorch	3.3–5.5	−0.6
	Output switch, model RLC SR-7C-H from RLC Electronics	0–18	−0.3
	Amplifier, model AMF-6D-00101800-35-20P from Miteq	0.1–18	42
E	Input switch, model RLC SR-6C-H from RLC Electronics	0–18	−0.3
	Band-pass cavity filter, 8IZ6-7200/R3600-S from Lorch	5.4–9	−0.4
	Output switch, model RLC SR-7C-H from RLC Electronics	0–18	−0.3
	Amplifier, model AMF-6D-00101800-35–20P from Miteq	0.1–18	42
**Common for all channels**	External spiral coaxial cable	0–18	−15

## Data Availability

Data are contained within the article.
